# The impact of catheter replacement in patients with catheter-associated urinary tract infection: study protocol paper for a multicentre, non-inferiority randomized controlled trial (REPLACE)

**DOI:** 10.1186/s12879-026-12890-x

**Published:** 2026-02-27

**Authors:** Eline Schippers, Janneke van Uhm, Merel Wilmsen, Anke Tigchelaar, Wouter Rozemeijer, Barbara Schout, Elske van den Akker-van Marle, Martha van der Beek, Maxime Kummeling, Rolf Groenwold, Suzanne Geerlings, Merel Lambregts

**Affiliations:** 1 Leiden University Center of Infectious Diseases (LUCID), Leiden University Medical Center, Leiden, The Netherlands; 2 Department of Urology, Leiden University Medical Center, Leiden, The Netherlands; 3 Spinal Cord Injury Association, Nijkerk, The Netherlands; 4 Department of Medical Microbiology, Noordwest Ziekenhuisgroep, Alkmaar, The Netherlands; 5 Department of Urology, Alrijne Ziekenhuis, Leiderdorp, The Netherlands; 6 Department of Biomedical Data Sciences, Leiden University Medical Centre, Leiden, The Netherlands; 7 Department of Clinical Epidemiology, Leiden University Medical Center, Leiden, The Netherlands; 8 Department of Internal Medicine, Division of Infectious Diseases, Amsterdam University Medical Center, University of Amsterdam, Amsterdam, The Netherlands

**Keywords:** Urinary tract infection, Catheter associated urinary tract infection, Catheter, Catheter related infection, Urosepsis

## Abstract

**Background:**

Catheter-associated urinary tract infection (CAUTI) is the most common healthcare associated infection. Current guidelines state that catheters should be replaced in patients with CAUTI to hasten symptom resolution and to lower the risk of recurrence. However, the available literature is limited and there is significant practice variation globally. The aim of this study is to determine whether not changing the catheter during CAUTI treatment affects the recurrence-risk.

**Methods and analysis:**

The REPLACE study is a multicentre, non-inferiority- randomized controlled trial designed to assess whether retaining the urinary catheter in patients with CAUTI is non-inferior to replacement in terms of the risk of a recurrent CAUTI within 90 days. Secondary outcome measures include 30-day mortality, health-related quality of life, time to resolution of CAUTI symptoms, complications related to catheter replacement and healthcare and societal costs. Patients aged 18 years or older with an indwelling catheter (transurethral or suprapubic) and CAUTI are eligible. CAUTI is defined according to the Infectious Diseases Society of America (IDSA) criteria. Three hundred patient will be randomised 1:1 to catheter retention (intervention group) or replacement (control group). In the intervention group, the catheter is not replaced. In the control group the catheter will be replaced within 3 days after start of antibiotic therapy. The primary outcome (recurrent CAUTI within 90 days) will be assessed for non-inferiority (margin of 10%).

**Discussion:**

This trial will address a critical evidence gap and may inform future guidelines regarding catheter management in CAUTI. If replacement is found to have no benefit, this could reduce unnecessary interventions, lower healthcare costs and material use, contribute to sustainability, and lessen the burden on both patients and healthcare organizations.

**Trial registration:**

Registered at clinicaltrial.gov on 17 April 2025 with registration number: NCT06936631.

**Supplementary Information:**

The online version contains supplementary material available at 10.1186/s12879-026-12890-x.

## Background

Due to the widespread use of urinary catheters, catheter-associated urinary tract infection (CAUTI) is the most common healthcare-associated infection [1]. As urinary catheter use is expected to rise with an aging population, the number of CAUTIs will likely increase accordingly [2]. CAUTIs can result in serious, sometimes fatal complications, including urosepsis [3–6].

To fasten recovery and reduce the risk of recurrent CAUTI, current guidelines recommend to replace the catheter at the onset of CAUTI when the indwelling catheter has been in place for more than two weeks [1, 6, 7]. This recommendation, however, is based on limited evidence as only four conflicting studies have been identified that investigate catheter replacement during CAUTI [8]. Current guidelines rely on only one of these four studies which is a small randomized controlled trial (RCT) that favoured catheter replacement [9]. The study reported shorter recovery times and lower recurrence rates in the replacement group compared to the retention group. These findings were contradicted by a second RCT [10], which found higher recurrence rates in the replacement group. However, in that study, antimicrobial treatment was shorter in the replacement group. Two observational studies found no effect of catheter replacement on either recurrence or symptom duration [11, 12], highlighting the paucity of the current state of evidence on catheter management during CAUTI.

In light of the limited evidence, a survey has been conducted to assess clinical practice regarding catheter management in CAUTI (manuscript in preparation [13]). It revealed considerable variation in catheter management practices among urologists, microbiologists, and internists. Urologists indicated more often that they routinely retain the transurethral catheter, compared to internists and medical microbiologists. The primary reasons for catheter replacement were the fact that it is a guideline recommendation and the expectation that replacement would be beneficial from a pathophysiological rationale. From a theoretical perspective, catheter replacement aligns with the principle of source control, as removal of the catheter may eliminate biofilm-associated pathogens that can persist during antimicrobial therapy and contribute to recurrent CAUTI [14]. Main reasons for retaining the catheter were the lack of scientific evidence supporting clinical benefit and the risk of procedural complications of the procedure.

The REPLACE study aims to generate evidence to inform future recommendations for urinary catheter management in the treatment of CAUTI. We hypothesize that not changing the catheter during a CAUTI is non-inferior to replacing it with regard to the risk of recurrent CAUTI. This study therefore potentially contributes to reducing healthcare costs and sustainability by minimizing waste generated from catheter replacements, while also alleviating the workload on personnel when the procedure is redundant.

## Methods

### Study design and setting

This study is a multicentre, two-arm, randomized controlled non-inferiority trial. Patients will be recruited from two academic and fifteen non-academic hospitals across the Netherlands between April 2025 and February 2028. The number of participating centres may be increased depending on the recruitment rate.

### Participants

Recruitment will occur in both inpatient and outpatient settings.

#### Inclusion criteria

To be eligible to participate in this study, a subject must meet all of the following criteria:

- Aged ≥ 18 years.
- Indwelling transurethral or suprapubic catheter for at least 2 weeks.
- CAUTI according to the IDSA guideline criteria, i.e.,
  - At least one of the following symptoms: onset or worsening of fever (> 38 degrees), rigors, altered mental status, malaise or lethargy with no other identified cause, flank pain, costovertebral angle tenderness, acute haematuria, pelvic discomfort, or suprapubic pain/tenderness. In patients with spinal cord injury: increased spasticity, autonomic dysreflexia, or sense of unease.
  - Urine culture with ≥ 10^3^ colony-forming units (CFU)/mL of ≥ 1 bacterial species, or < 10^3^ CFU/mL along with a positive blood culture with the same microorganism as the urine culture.
- The ability to provide written informed consent.

Participants may be enrolled before culture results are available. Consequently, participants may be excluded after inclusion (and randomization) if they are later found not to meet the microbiological criterion.

#### Exclusion criteria

A potential participant who meets any of the following criteria will be excluded from participation in this study:

- Antibiotic treatment initiated more than 3 days prior to inclusion.
- Immunodeficiency: High-dose corticosteroid use (any equivalent of ≥ 20 mg prednisolone per day for > 4 weeks), severe primary immunodeficiency, organ transplant, neutropenia (absolute neutrophil count < 0.50 × 10⁹/L).
- Discontinuation of the indication for the indwelling catheter.
- A planned (routine) catheter replacement during antibiotic therapy.
- Contraindications for catheter replacement.
- Kidney catheters (nephrostomy or double-J catheter).
- Needing continuous bladder irrigations because of gross haematuria.
- Bladder stones.
- Pregnancy.
- A life expectancy of less than 90 days, as estimated by the treating physician.

### Study interventions

Patients will be assessed for CAUTI according to standard practice of care. Potential participants will be screened against inclusion/exclusion criteria by the central study team. Eligible patients will receive written study information and provide written or remote informed consent before randomization.

After obtaining informed consent, participants will be randomized to either catheter replacement (control group) or catheter retention (intervention group). In the replacement group, the catheter will be replaced within 3 days of starting antibiotic treatment. All patients (men and women) will receive a 10-day course of antibiotics. The choice of antibiotic agent is at the discretion of the treating physician, except for nitrofurantoin, fosfomycin, and trimethoprim as these may be considered inferior for the treatment of CAUTI with systemic symptoms. Once culture and susceptibility results are available, therapy must be adjusted according to the EUCAST susceptibility criteria.

### Randomization

Patients will be allocated to the control or intervention group using the Castor Electronic Data Capture (EDC) system, in a 1:1 ratio. Stratified randomization will not be applied, as no single dominant prognostic factor for recurrence is known.

### Data collection

Patients will be followed for a total period of 180 days, with seven telephone visits (Table 1). All follow-ups are done by a member of the central study team at the LUMC. Data will be collected on patient characteristics, symptoms, and urine analyses, and several questionnaires will be administered.

#### Clinical data

At baseline, the following patient characteristics will be recorded: gender, age, height, weight, vital signs, social circumstances, medical history, medication use, catheter specific data (type of catheter, duration). The length of hospital stay, intensive care unit (ICU) admission during hospitalization, and type and treatment duration of antimicrobial agent(s) will additionally be collected.

Upon inclusion, CAUTI symptoms will be recorded, including onset or worsening of fever (> 38 degrees), rigors, altered mental status, malaise or lethargy with no other identified cause, flank pain, costovertebral angle tenderness, acute haematuria, pelvic discomfort, or suprapubic pain/tenderness. In patients with spinal cord injury increased spasticity, autonomic dysreflexia, or sense of unease will be assessed. Participants will be provided with a paper diary to record their symptoms for the first 14 days after start of antibiotic treatment. During each follow-up visit, CAUTI symptoms will be reassessed to track symptom duration and identify possible CAUTI recurrences.

#### Laboratory data

Urine samples will be collected according to standard of care for microbiological culture. Uropathogens growing at ≥ 10³ CFU/mL will be identified and subjected to susceptibility testing according to standard laboratory protocols. If obtained as part of routine clinical care, blood culture results, inflammation parameters, kidney function and lactate will be collected from the medical records.

#### Questionnaires

Questionnaires will be administered at intermittent timepoints (Table 1). The PROMIS and EQ-5D-5 L will be used to assess health-related quality of life [15, 16]. Healthcare and societal costs will be evaluated using a selection of items relevant for this population of the medical consumption (iMCQ) and productivity (iPCQ) questionnaire [17]. If patients are able to complete questionnaires digitally, questionnaires are sent to the patient via Castor EDC.

**Table 1:** An overview of the study visits and questionnaires administered during each visit

	Day 1	Day 3	Day 8	Day 15	Day 29	Day 60	Day 90	Day 180
CAUTI symptoms								
PROMIS								
EQ-5D-5 L								
iMCQ								
iPCQ								

### Outcome measures

The primary outcome is a recurrent CAUTI within 90 days after completion of antibiotic treatment, defined according to the same IDSA-based clinical and microbiological criteria used for inclusion. The pathogen does not have to be identical to the causative pathogen that was identified during the first episode.

All participants are instructed to contact the central study team should they experience CAUTI symptoms. Additionally, the physicians at the participating centres and general practitioners are instructed to report potential recurrences among participants.

Secondary outcomes:

- Patient-related outcomes: 30-day mortality, health-related quality of life assessed using the PROMIS and EQ-5D-5L questionnaires.
- Process-related outcomes: time to resolution of CAUTI symptoms, time to resolution of fever, complications of catheter replacement and severity of the complications (5-level Likert scale).
- Resource-related outcomes: length of hospital stay, ICU-admission during hospital stay, healthcare and societal cost using a selection of items relevant for this population of the medical consumption (iMCQ) and productivity (iPCQ) questionnaire.

### Statistical analysis

#### Sample size

It is planned to recruit 300 participants (150 in each treatment arm). For both groups, we assume a recurrence rate of 10% [18] within 90 days after start of antibiotic therapy. Analyzing the data as a non-inferiority study, taking a non-inferiority margin of 10%, and a (one-sided) type-I error rate (alpha) of 2.5%, a study with 300 participants would have a power of approximately 80%. A power of 80% would indicate that the likelihood is 80% that the upper-limit of the 95% confidence interval for the 90-day risk difference will exclude a difference of 10% in favour of the control (replacement) group. To account for post-randomization exclusions, we plan to randomize 400 participants.

An interim analysis of the overall recurrence rate will be performed after 50% of the initially planned sample size has completed 90 days of follow-up. The interim analysis will not assess treatment effectiveness, and no early stopping for efficacy or futility is planned. The sole purpose of this interim analysis is to evaluate whether the observed overall recurrence rate aligns with the assumptions used for the sample size calculation. If the recurrence rate is found to be below 7.5%, the project group will consider whether an increase in the sample size is warranted.

#### Analyses of outcome measures

Descriptive statistics will be used to summarize baseline characteristics. In the primary analysis, the risk difference with (two-sided) 95% confidence interval will be computed to compare the differences in 90-day recurrence risk between the two treatment arms, in both the intention-to-treat population and the per-protocol population. Regarding the primary analysis, multiple imputation will be used to deal with missing outcome data (i.e. missing information regarding CAUTI recurrence). For the primary outcome, three sensitivity analyses will be performed to account for missing data. In the first sensitivity analysis, a complete case analysis will be performed. Additionally, a worst-case and best-case scenario analysis will be performed, in which patients who drop out of the study before 90 days will be included under the assumption that they experienced a recurrence (worst-case scenario) or no recurrence (best-case scenario), respectively.

The primary analysis will not take patient characteristics into account, as these are expected to be balanced between treatment groups (by randomization). In a secondary analysis, multivariable analysis will be performed including variables that are associated with recurrence of CAUTI.

The secondary outcome time-to-resolution will be analysed using Kaplan Meier survival analysis. For patient-related outcomes measures, such as PROMIS and EQ-5D-5 L, repeated measures analyses will be performed to compare these outcomes between the groups. For these outcome measures, multiple imputation will be used to deal with missing outcome data. Process and resource related outcome measures will be analysed in the per-protocol population. In addition, a cost-effectiveness analysis from a societal perspective with a 6-month time horizon and a budget impact analysis will be performed.

### Patient involvement

Patient involvement has been embedded in all phases of this study. The study design was developed in co-creation with a patient representative to ensure relevance and patient-centeredness. In addition, a UTI patient panel has provided structured feedback on patient information materials, perceived burden of study procedures, and potential barriers to participation. During the trial, enrolled participants will be invited to share their experiences through short surveys and focus groups to improve recruitment and patient-facing materials.

### Stakeholder and co-creation involvement

In addition to patient involvement, the study design was developed through a structured co-creation process involving a broad range of stakeholders. Representatives from national healthcare insurers, Knowledge Institute of the Dutch Association of Medical Specialists, the National Health Care Institute, and the professional associations for nursing, microbiology, infectious diseases, and urology participated in the co-creation phase. These stakeholders contributed to the selection of clinically meaningful outcomes, the operational feasibility of study procedures, the relevance and acceptability of the non-inferiority margin, and the anticipated implications for clinical practice and healthcare systems. Their input ensured that the study design reflects both clinical and societal perspectives and aligns with current implementation needs.

### Observational cohort

In addition to the RCT, an observational cohort will be established for adult patients with CAUTI who either decline participation in the intervention study or are ineligible based on the inclusion and exclusion criteria. These patients will be informed about the observational cohort and will be included only after providing written (or remote) informed consent. Baseline data and outcome measures will be extracted from the electronic medical record, and participants will be contacted once at 90 days by a member of the central study team at the LUMC to assess outcomes. Questionnaires and symptom diaries will not be collected in the observational cohort.

The observational cohort is included to enhance external validity and to provide insight into the characteristics and outcomes of patients who are not represented in the randomized trial. This allows assessment of potential selection and non-participation bias and enables comparison of baseline characteristics and clinical outcomes between trial participants and non-participants. In addition, the observational cohort will provide descriptive data on CAUTI management and outcomes in routine clinical practice, thereby facilitating interpretation and contextualization of the RCT findings.

## Discussion

The REPLACE study outlined in this protocol is the first RCT with sufficient power to investigate the effect of catheter replacement in CAUTI. Even though CAUTIs are the most common healthcare-associated infection, current recommendations for catheter management during treatment are based on limited and conflicting evidence [8]. As a result of this evidence gap, considerable practice variation exists, underlining the need for this RCT to inform the guidelines and standardize practice.

### Strengths and limitations

One of the primary strengths of this study is its randomized, multicentre design, which enhances internal validity and generalizability. The planned sample size provides sufficient power to evaluate non-inferiority regarding recurrent CAUTI. In addition to traditional clinical outcomes, this trial includes patient-reported outcome measures, and a full economic evaluation, thereby capturing the impact of catheter management strategies on patients and healthcare systems alike. Another important strength is the extensive co-creation process. Patients, alongside experts from all relevant scientific associations (urology, infectious diseases and medical microbiology) were actively involved in shaping the study design. Their input was crucial in selecting relevant outcomes, the acceptability and feasibility of study procedures, and defining the clinically relevant non-inferiority margin. This collaborative approach increases the methodological robustness and the clinical relevance of the trial, and facilitates the implementation phase of the results. Additionally, the consistent antimicrobial therapy between both groups is a strength. Furthermore, the study adheres to the IDSA guideline definition of CAUTI, which incorporates both symptoms and urine culture results. This definition is consistently applied throughout the study, in both the inclusion criteria and recurrences, ensuring diagnostic consistency.

However, several limitations should be acknowledged. First, the inability to blind participants and clinicians may introduce reporting bias in symptom resolution and the reporting of recurrences. The risk of differential reporting is mitigated by the use of predefined symptom definitions, structured questionnaires, and centralised follow-up by a study team independent of clinical decision-making. A second challenge will be recruitment, as both patients and clinicians may have pre-existing preferences regarding catheter management and may not be willing to be randomized. To ensure adequate recruitment, additional study sites may be added if required. The observational cohort will allow us to monitor characteristics of patients who decline participation, thereby maintaining insight into potential non-participation bias.

Another limitation is the exclusion of potential participants who lack decision-making capacity (such as patients with delirium, dementia, or an intellectual disability), as the current inclusion criteria state that all participants must be able to provide informed consent. A substantial proportion of patients presenting with a CAUTI experience delirium or have pre-existing impaired decision-making capacity. Excluding these patients may therefore introduce selection bias and limit the generalisability of the study findings. An amendment in order to facilitate the participation of these patients is in preparation.

A further limitation is the potential for misclassification bias, which is well recognized in CAUTI research. Despite the use of the IDSA definition combining clinical and microbiological criteria, CAUTI remains a diagnostically challenging entity, and some degree of misclassification cannot be entirely excluded.

Lastly, the study population is limited to adults with a transurethral or suprapubic catheter. Patients with nephrostomy tubes or double-J stents are excluded, even though replacement is particularly burdensome in these populations and evidence is virtually absent. The findings of the REPLACE study cannot be directly extrapolated to kidney catheters, but they may provide a rationale for future studies investigating whether the conclusions of this trial hold true in these settings.

### Implications and future directions

Current national and international guidelines, advice to change the catheter with a low level of evidence [1, 7, 8]. If this trial demonstrate that catheter retention is non-inferior to replacement, clinical practice could be standardized accordingly in future guideline updates. This may reduce the number of invasive procedures, decrease healthcare costs, lower material waste and alleviate patient burden without compromising safety. Conversely, if catheter retention is not found to be non-inferior, the results will strengthen the evidence base supporting current recommendations for catheter replacement and may improve adherence in clinical practice.

## Supplementary Information

Below is the link to the electronic supplementary material.

Supplementary Material 1: SPIRIT checklist

## Data Availability

No datasets were generated or analysed during the current study.

## Declarations

### Trial status

Recruitment began in April 2025 and is expected to be completed by February 2028. Up to now, 45 participants are included.

## Abbreviations

Castor EDC: Castor Electronic Data Capture (EDC)
CAUTI: Catheter-associated urinary tract infection
CFU: Colony-forming units
EQ-5D-5L: The 5-level EQ-5D version introduced by the EuroQol Group
EUCAST: European Committee on Antimicrobial Susceptibility Testing
ICU: Intensive care unit
IDSA: Infectious Diseases Society of America
iMCQ: The iMTA Medical Consumption Questionnaire
iPCQ: The iMTA Productivity Cost Questionnaire
LUMC: Leiden University Medical Centre
PROMIS: Patient-Reported Outcomes Measurement Information System
RCT: Randomized controlled trial
UTI: Urinary tract infection

## References

[1] Hooton TM, Bradley SF, Cardenas DD, Colgan R, Geerlings SE, Rice JC, et al. Diagnosis, prevention, and treatment of catheter-associated urinary tract infection in adults: 2009 international clinical practice guidelines from the infectious diseases society of America. Clin Infect Dis. 2010;50(5):625–63. 20175247 10.1086/650482

[2] Berendsen SA, van Doorn T, Blok BFM. Urinary catheterization from 1997 to 2018: a Dutch population-based cohort. Ther Adv Urol. 2021;13:17562872211007625. 33912247 10.1177/17562872211007625 PMC8047834

[3] Rudman D, Hontanosas A, Cohen Z, Mattson DE. Clinical correlates of bacteremia in a veterans administration extended care facility. J Am Geriatr Soc. 1988;36(8):726–32. 3042843 10.1111/j.1532-5415.1988.tb07175.x

[4] Jepsen OB, Larsen SO, Dankert J, Daschner F, Gronroos P, Meers PD, et al. Urinary-tract infection and bacteraemia in hospitalized medical patients–a European multicentre prevalence survey on nosocomial infection. J Hosp Infect. 1982;3(3):241–52. 6183317 10.1016/0195-6701(82)90043-3

[5] Melzer M, Welch C. Does the presence of a urinary catheter predict severe sepsis in a bacteraemic cohort? J Hosp Infect. 2017;95(4):376–82. 28202189 10.1016/j.jhin.2017.01.003

[6] Bonkat G, Kranz J, Cai T, Geerlings SE, Köves B, Pilatz A et al. EAU Guidelines on Urological Infections 2025 [Available from: https://uroweb.org/guidelines/urological-infections

[7] Terpstra ML, Geerlings SE, van Nieuwkoop C, van Haarst EP, Boom H, Knottnerus BJ, et al. Optimization of the antibiotic policy in the netherlands: SWAB guidelines for antimicrobial therapy of urinary tract infections in adults. Leiden: Stichting Werkgroep Antibioticabeleid; 2020.

[8] Westgeest AC, van Uhm JIM, Pattacini L, Rozemeijer W, Schout BMA, Groenwold RHH, et al. Catheter replacement in catheter-associated urinary tract infection: current state of evidence. Eur J Clin Microbiol Infect Dis. 2024;43(8):1631–7. 38916643 10.1007/s10096-024-04878-9 PMC11271365

[9] Raz R, Schiller D, Nicolle LE. Chronic indwelling catheter replacement before antimicrobial therapy for symptomatic urinary tract infection. J Urol. 2000;164(4):1254–8. 10992375

[10] Darouiche RO, Al Mohajer M, Siddiq DM, Minard CG. Short versus long course of antibiotics for catheter-associated urinary tract infections in patients with spinal cord injury: a randomized controlled noninferiority trial. Arch Phys Med Rehabil. 2014;95(2):290–6. 24035770 10.1016/j.apmr.2013.09.003

[11] Kumazawa J, Matsumoto T. The dipstick test in the diagnosis of UTI and the effect of pretreatment catheter exchange in catheter-associated UTI. Infection. 1992;20(Suppl 3):S157–9. 1490742 10.1007/BF01704360

[12] Babich T, Zusman O, Elbaz M, Ben-Zvi H, Paul M, Leibovici L, et al. Replacement of urinary catheter for urinary tract infections: A prospective observational study. J Am Geriatr Soc. 2018;66(9):1779–84. 30094820 10.1111/jgs.15517

[13] Schippers EC, van Uhm JIM, Geerlings SE, Lambregts MMC. To change or not to change: clinical practice variation in catheter replacement for catheter-associated urinary tract infections. Paper poster (nr. P3092) at ESCMID global 2025.

[14] Kass E. Asymptomatic infections of the urinary tract. Trans Assoc Am Physicians. 1956;69:56–64. 13380946

[15] Elsman EBM, Roorda LD, Crins MHP, Boers M, Terwee CB. Dutch reference values for the Patient-Reported outcomes measurement information system scale v1.2 - Global health (PROMIS-GH). J Patient Rep Outcomes. 2021;5(1):38. 33978855 10.1186/s41687-021-00314-0 PMC8116369

[16] EQ-5D 2025 EuroQol Research Foundation. [Available from: https://euroqol.org/register/]

[17] IMCQ IPCQ questionnaires. [Available from: https://www.imta.nl/questionnaires/]

[18] Gomila A, Carratala J, Eliakim-Raz N, Shaw E, Tebe C, Wolkewitz M, et al. Clinical outcomes of hospitalised patients with catheter-associated urinary tract infection in countries with a high rate of multidrug-resistance: the COMBACTE-MAGNET RESCUING study. Antimicrob Resist Infect Control. 2019;8:198. 31827779 10.1186/s13756-019-0656-6 PMC6892205

